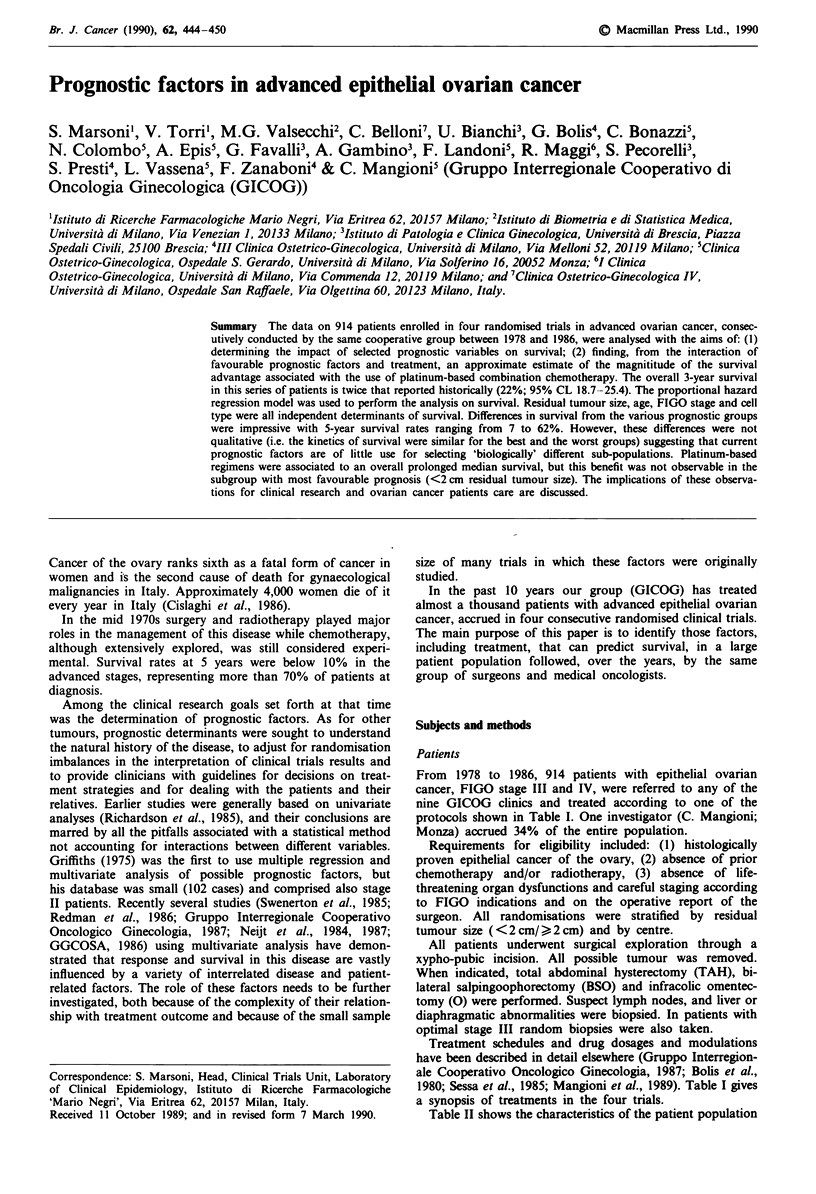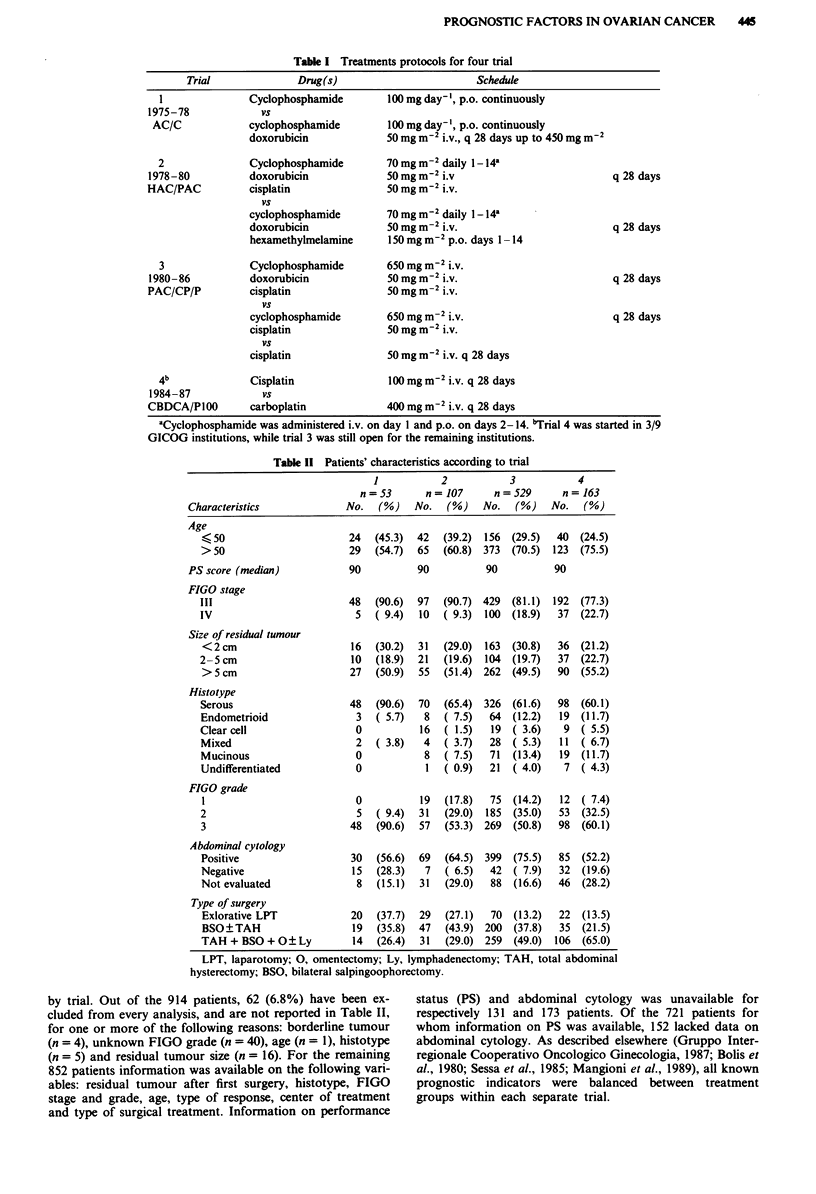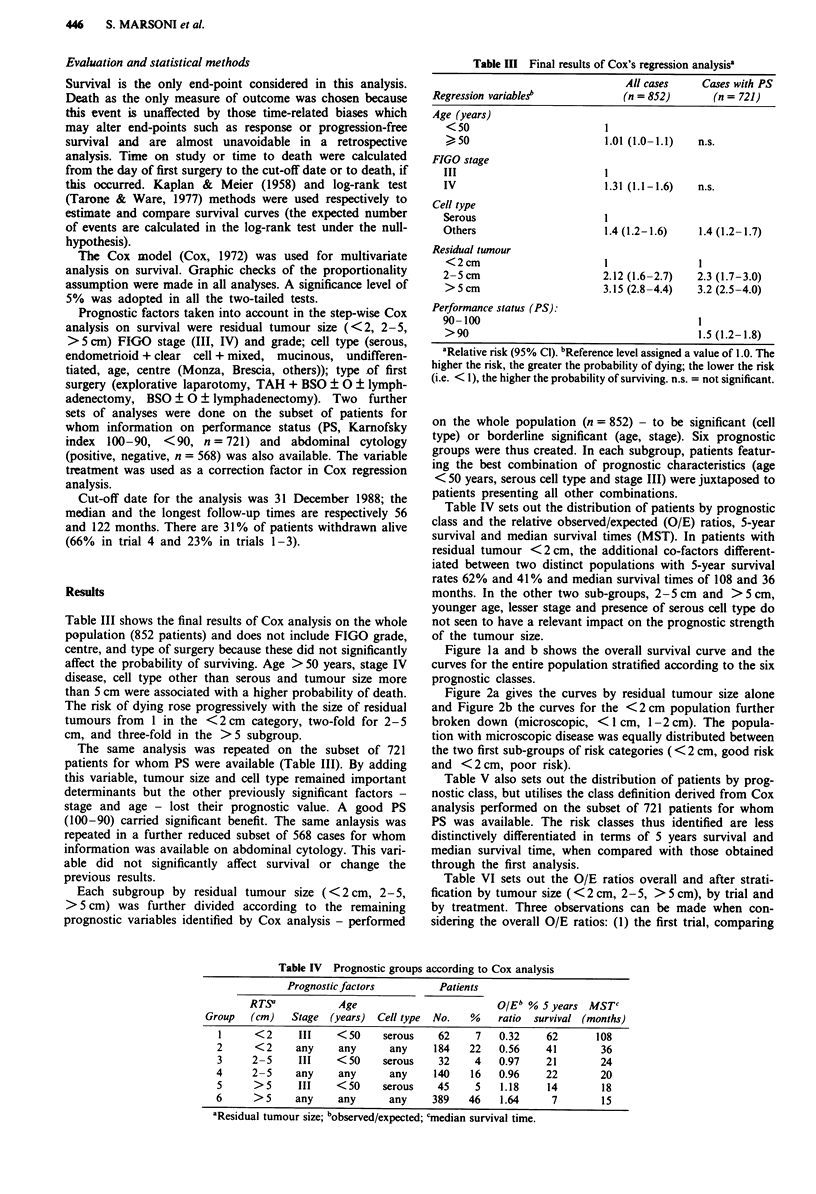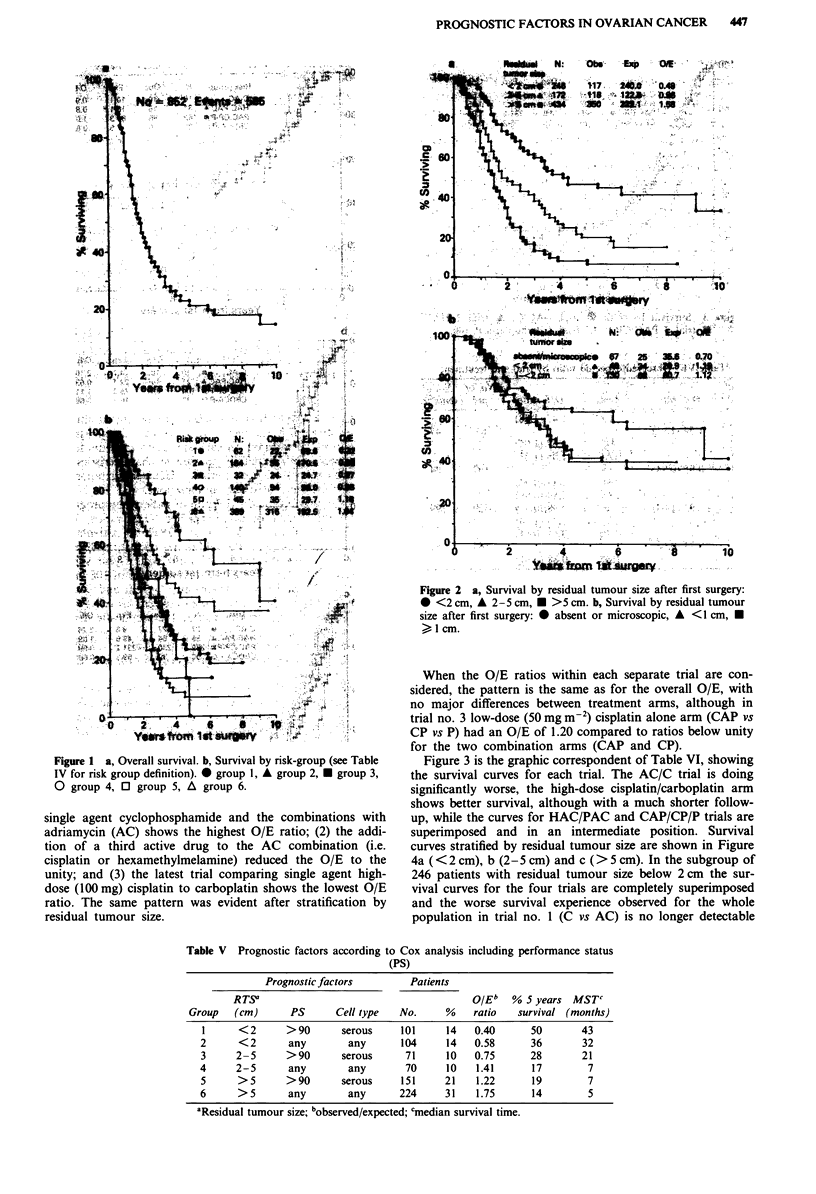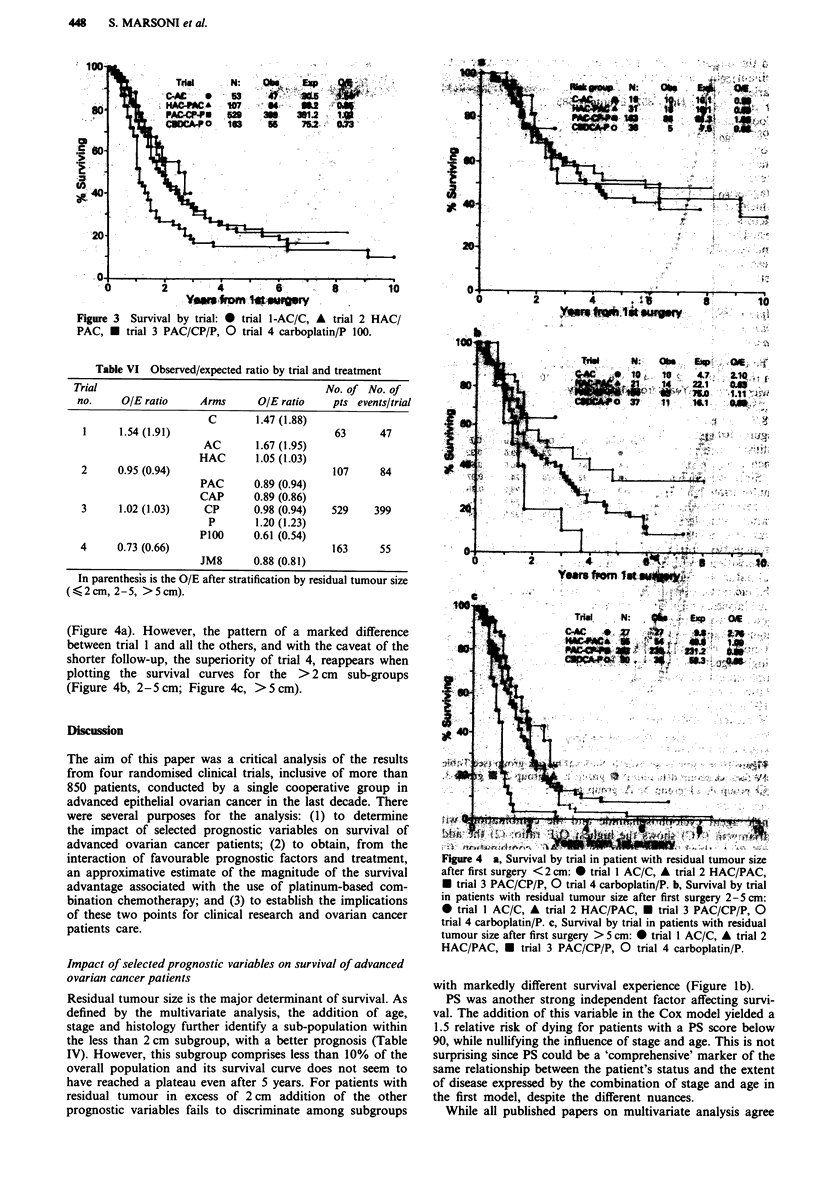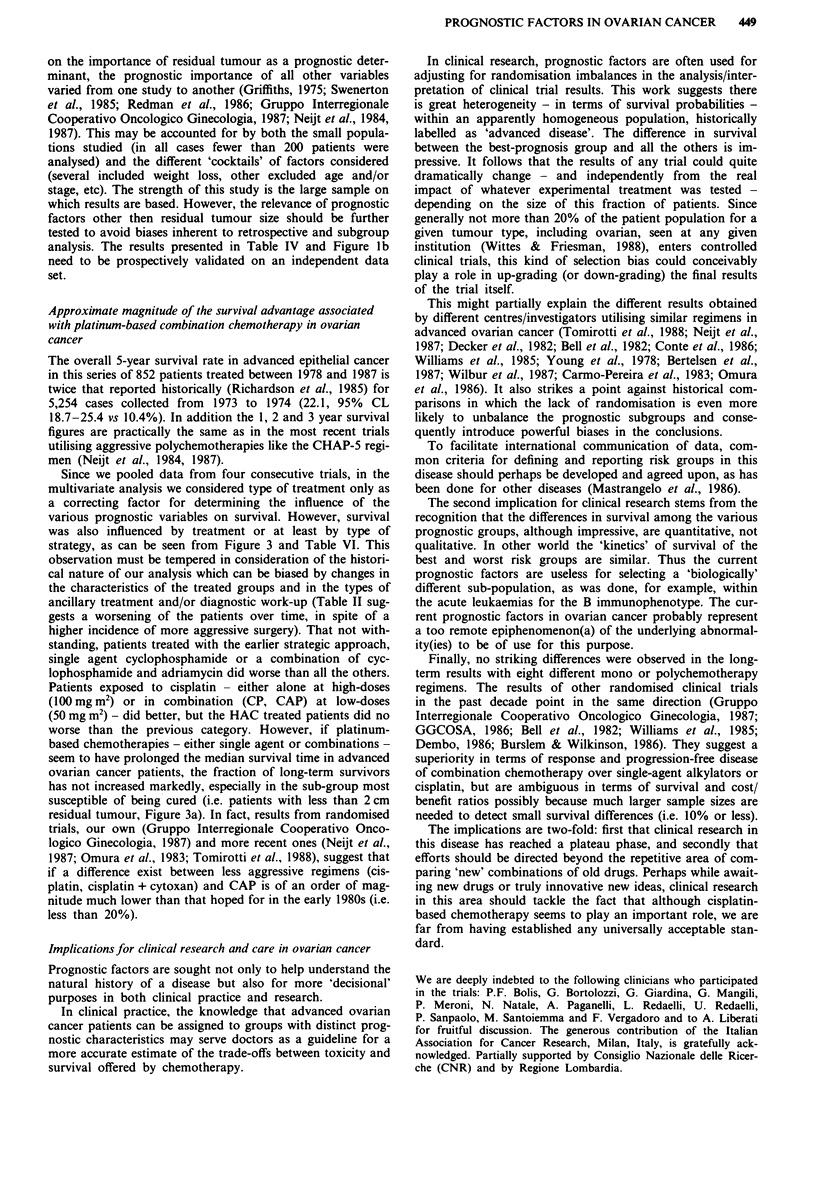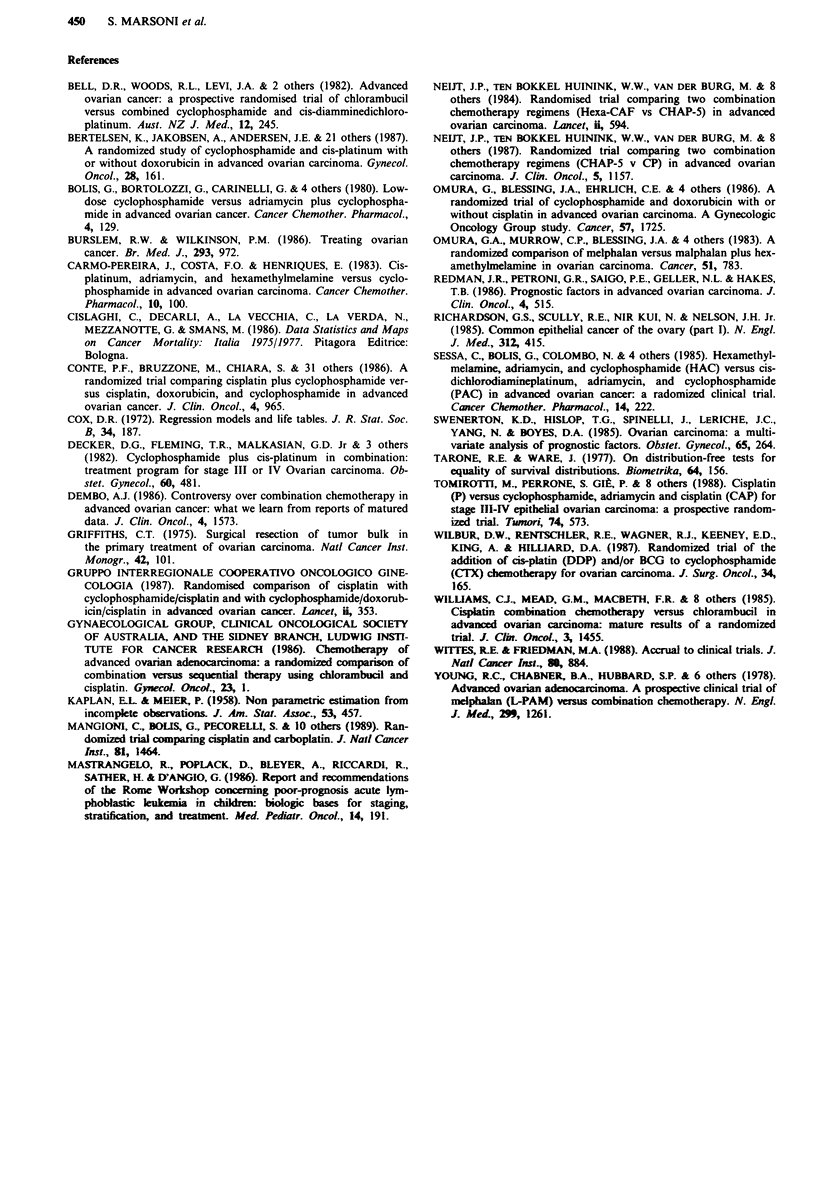# Prognostic factors in advanced epithelial ovarian cancer. (Gruppo Interregionale Cooperativo di Oncologia Ginecologica (GICOG)).

**DOI:** 10.1038/bjc.1990.315

**Published:** 1990-09

**Authors:** S. Marsoni, V. Torri, M. G. Valsecchi, C. Belloni, U. Bianchi, G. Bolis, C. Bonazzi, N. Colombo, A. Epis, G. Favalli

**Affiliations:** Istituto di Ricerche Farmacologiche Mario Negri, Milano, Italy.

## Abstract

The data on 914 patients enrolled in four randomised trials in advanced ovarian cancer, consecutively conducted by the same cooperative group between 1978 and 1986, were analysed with the aims of: (1) determining the impact of selected prognostic variables on survival; (2) finding, from the interaction of favourable prognostic factors and treatment, an approximate estimate of the magnitude of the survival advantage associated with the use of platinum-based combination chemotherapy. The overall 3-year survival in this series of patients is twice that reported historically (22%; 95% CL 18.7-25.4). The proportional hazard regression model was used to perform the analysis on survival. Residual tumour size, age, FIGO stage and cell type were all independent determinants of survival. Differences in survival from the various prognostic groups were impressive with 5-year survival rates ranging from 7 to 62%. However, these differences were not qualitative (i.e. the kinetics of survival were similar for the best and the worst groups) suggesting that current prognostic factors are of little use for selecting 'biologically' different sub-populations. Platinum-based regimens were associated to an overall prolonged median survival, but this benefit was not observable in the subgroup with most favourable prognosis (less than 2 cm residual tumour size). The implications of these observations for clinical research and ovarian cancer patients care are discussed.


Br. J. Cancer (1990), 62, 444–450 © Macmillan Press Ltd., 1990

### Prognostic factors in advanced epithelial ovarian cancer

S. Marsoni[1], V. Torri[1], M.G. Valsecchi[2], C. Belloni[7], U. Bianchi[3], G. Bolis[4], C. Bonazzi[5], N. Colombo[5], A. Epis[5], G. Favalli[3], A. Gambino[3], F. Landoni[5], R. Maggi[6], S. Pecorelli[3], S. Presti[4], L. Vassena[5], F. Zanaboni[4] & C. Mangioni[5] (Gruppo Interregionale Cooperativo di Oncologia Ginecologica (GICOG))

[1]*Istituto di Ricerche Farmacologiche Mario Negri, Via Eritrea 62, 20157 Milano;* [2]*Istituto di Biometria e di Statistica Medica, Università di Milano, Via Venezian 1, 20133 Milano;* [3]*Istituto di Patologia e Clinica Ginecologica, Università di Brescia, Piazza Spedali Civili, 25100 Brescia;* [4]*III Clinica Ostetrico-Ginecologica, Università di Milano, Via Melloni 52, 20119 Milano;* [5]*Clinica Ostetrico-Ginecologica, Ospedale S. Gerardo, Università di Milano, Via Solferino 16, 20052 Monza;* [6]*I Clinica Ostetrico-Ginecologica, Università di Milano, Via Commenda 12, 20119 Milano; and* [7]*Clinica Ostetrico-Ginecologica IV, Università di Milano, Ospedale San Raffaele, Via Olgettina 60, 20123 Milano, Italy.*

**Summary** The data on 914 patients enrolled in four randomised trials in advanced ovarian cancer, consecutively conducted by the same cooperative group between 1978 and 1986, were analysed with the aims of: (1) determining the impact of selected prognostic variables on survival; (2) finding, from the interaction of favourable prognostic factors and treatment, an approximate estimate of the magnititude of the survival advantage associated with the use of platinum-based combination chemotherapy. The overall 3-year survival in this series of patients is twice that reported historically (22%; 95% CL 18.7–25.4). The proportional hazard regression model was used to perform the analysis on survival. Residual tumour size, age, FIGO stage and cell type were all independent determinants of survival. Differences in survival from the various prognostic groups were impressive with 5-year survival rates ranging from 7 to 62%. However, these differences were not qualitative (i.e. the kinetics of survival were similar for the best and the worst groups) suggesting that current prognostic factors are of little use for selecting 'biologically' different sub-populations. Platinum-based regimens were associated to an overall prolonged median survival, but this benefit was not observable in the subgroup with most favourable prognosis (<2 cm residual tumour size). The implications of these observations for clinical research and ovarian cancer patients care are discussed.

Cancer of the ovary ranks sixth as a fatal form of cancer in women and is the second cause of death for gynaecological malignancies in Italy. Approximately 4,000 women die of it every year in Italy (Cislaghi *et al.*, 1986).

In the mid 1970s surgery and radiotherapy played major roles in the management of this disease while chemotherapy, although extensively explored, was still considered experimental. Survival rates at 5 years were below 10% in the advanced stages, representing more than 70% of patients at diagnosis.

Among the clinical research goals set forth at that time was the determination of prognostic factors. As for other tumours, prognostic determinants were sought to understand the natural history of the disease, to adjust for randomisation imbalances in the interpretation of clinical trials results and to provide clinicians with guidelines for decisions on treatment strategies and for dealing with the patients and their relatives. Earlier studies were generally based on univariate analyses (Richardson *et al.*, 1985), and their conclusions are marred by all the pitfalls associated with a statistical method not accounting for interactions between different variables. Griffiths (1975) was the first to use multiple regression and multivariate analysis of possible prognostic factors, but his database was small (102 cases) and comprised also stage II patients. Recently several studies (Swenerton *et al.*, 1985; Redman *et al.*, 1986; Gruppo Interregionale Cooperativo Oncologico Ginecologia, 1987; Neijt *et al.*, 1984, 1987; GGCOSA, 1986) using multivariate analysis have demonstrated that response and survival in this disease are vastly influenced by a variety of interrelated disease and patient-related factors. The role of these factors needs to be further investigated, both because of the complexity of their relationship with treatment outcome and because of the small sample size of many trials in which these factors were originally studied.

In the past 10 years our group (GICOG) has treated almost a thousand patients with advanced epithelial ovarian cancer, accrued in four consecutive randomised clinical trials. The main purpose of this paper is to identify those factors, including treatment, that can predict survival, in a large patient population followed, over the years, by the same group of surgeons and medical oncologists.

### Subjects and methods

### *Patients*

From 1978 to 1986, 914 patients with epithelial ovarian cancer, FIGO stage III and IV, were referred to any of the nine GICOG clinics and treated according to one of the protocols shown in Table I. One investigator (C. Mangioni; Monza) accrued 34% of the entire population.

Requirements for eligibility included: (1) histologically proven epithelial cancer of the ovary, (2) absence of prior chemotherapy and/or radiotherapy, (3) absence of life-threatening organ dysfunctions and careful staging according to FIGO indications and on the operative report of the surgeon. All randomisations were stratified by residual tumour size (<2 cm/⩾2 cm) and by centre.

All patients underwent surgical exploration through a xypho-pubic incision. All possible tumour was removed. When indicated, total abdominal hysterectomy (TAH), bilateral salpingoophorectomy (BSO) and infracolic omentectomy (O) were performed. Suspect lymph nodes, and liver or diaphragmatic abnormalities were biopsied. In patients with optimal stage III random biopsies were also taken.

Treatment schedules and drug dosages and modulations have been described in detail elsewhere (Gruppo Interregionale Cooperativo Oncologico Ginecologia, 1987; Bolis *et al.*, 1980; Sessa *et al.*, 1985; Mangioni *et al.*, 1989). Table I gives a synopsis of treatments in the four trials.

Table II shows the characteristics of the patient population

Correspondence: S. Marsoni, Head, Clinical Trials Unit, Laboratory of Clinical Epidemiology, Istituto di Ricerche Farmacologiche 'Mario Negri', Via Eritrea 62, 20157 Milan, Italy.
Received 11 October 1989; and in revised form 7 March 1990.

**Table I** Treatments protocols for four trial

| *Trial* | *Drug(s)* | *Schedule* | |
|---|---|---|---|
| 1 | Cyclophosphamide | 100 mg day$^{-1}$, p.o. continuously | |
| 1975–78 | *vs* | | |
| AC/C | cyclophosphamide | 100 mg day$^{-1}$, p.o. continuously | |
| | doxorubicin | 50 mg m$^{-2}$ i.v., q 28 days up to 450 mg m$^{-2}$ | |
| 2 | Cyclophosphamide | 70 mg m$^{-2}$ daily 1–14[a] | |
| 1978–80 | doxorubicin | 50 mg m$^{-2}$ i.v | q 28 days |
| HAC/PAC | cisplatin | 50 mg m$^{-2}$ i.v. | |
| | *vs* | | |
| | cyclophosphamide | 70 mg m$^{-2}$ daily 1–14[a] | |
| | doxorubicin | 50 mg m$^{-2}$ i.v. | q 28 days |
| | hexamethylmelamine | 150 mg m$^{-2}$ p.o. days 1–14 | |
| 3 | Cyclophosphamide | 650 mg m$^{-2}$ i.v. | |
| 1980–86 | doxorubicin | 50 mg m$^{-2}$ i.v. | q 28 days |
| PAC/CP/P | cisplatin | 50 mg m$^{-2}$ i.v. | |
| | *vs* | | |
| | cyclophosphamide | 650 mg m$^{-2}$ i.v. | q 28 days |
| | cisplatin | 50 mg m$^{-2}$ i.v. | |
| | *vs* | | |
| | cisplatin | 50 mg m$^{-2}$ i.v. q 28 days | |
| 4[b] | Cisplatin | 100 mg m$^{-2}$ i.v. q 28 days | |
| 1984–87 | *vs* | | |
| CBDCA/P100 | carboplatin | 400 mg m$^{-2}$ i.v. q 28 days | |

[a]Cyclophosphamide was administered i.v. on day 1 and p.o. on days 2–14. [b]Trial 4 was started in 3/9 GICOG institutions, while trial 3 was still open for the remaining institutions.

**Table II** Patients' characteristics according to trial

| | *1* | | *2* | | *3* | | *4* | |
|---|---|---|---|---|---|---|---|---|
| | *n = 53* | | *n = 107* | | *n = 529* | | *n = 163* | |
| *Characteristics* | *No.* | *(%)* | *No.* | *(%)* | *No.* | *(%)* | *No.* | *(%)* |
| *Age* | | | | | | | | |
| ⩽50 | 24 | (45.3) | 42 | (39.2) | 156 | (29.5) | 40 | (24.5) |
| >50 | 29 | (54.7) | 65 | (60.8) | 373 | (70.5) | 123 | (75.5) |
| *PS score (median)* | 90 | | 90 | | 90 | | 90 | |
| *FIGO stage* | | | | | | | | |
| III | 48 | (90.6) | 97 | (90.7) | 429 | (81.1) | 192 | (77.3) |
| IV | 5 | ( 9.4) | 10 | ( 9.3) | 100 | (18.9) | 37 | (22.7) |
| *Size of residual tumour* | | | | | | | | |
| <2 cm | 16 | (30.2) | 31 | (29.0) | 163 | (30.8) | 36 | (21.2) |
| 2–5 cm | 10 | (18.9) | 21 | (19.6) | 104 | (19.7) | 37 | (22.7) |
| >5 cm | 27 | (50.9) | 55 | (51.4) | 262 | (49.5) | 90 | (55.2) |
| *Histotype* | | | | | | | | |
| Serous | 48 | (90.6) | 70 | (65.4) | 326 | (61.6) | 98 | (60.1) |
| Endometrioid | 3 | ( 5.7) | 8 | ( 7.5) | 64 | (12.2) | 19 | (11.7) |
| Clear cell | 0 | | 16 | ( 1.5) | 19 | ( 3.6) | 9 | ( 5.5) |
| Mixed | 2 | ( 3.8) | 4 | ( 3.7) | 28 | ( 5.3) | 11 | ( 6.7) |
| Mucinous | 0 | | 8 | ( 7.5) | 71 | (13.4) | 19 | (11.7) |
| Undifferentiated | 0 | | 1 | ( 0.9) | 21 | ( 4.0) | 7 | ( 4.3) |
| *FIGO grade* | | | | | | | | |
| 1 | 0 | | 19 | (17.8) | 75 | (14.2) | 12 | ( 7.4) |
| 2 | 5 | ( 9.4) | 31 | (29.0) | 185 | (35.0) | 53 | (32.5) |
| 3 | 48 | (90.6) | 57 | (53.3) | 269 | (50.8) | 98 | (60.1) |
| *Abdominal cytology* | | | | | | | | |
| Positive | 30 | (56.6) | 69 | (64.5) | 399 | (75.5) | 85 | (52.2) |
| Negative | 15 | (28.3) | 7 | ( 6.5) | 42 | ( 7.9) | 32 | (19.6) |
| Not evaluated | 8 | (15.1) | 31 | (29.0) | 88 | (16.6) | 46 | (28.2) |
| *Type of surgery* | | | | | | | | |
| Exlorative LPT | 20 | (37.7) | 29 | (27.1) | 70 | (13.2) | 22 | (13.5) |
| BSO±TAH | 19 | (35.8) | 47 | (43.9) | 200 | (37.8) | 35 | (21.5) |
| TAH + BSO + O±Ly | 14 | (26.4) | 31 | (29.0) | 259 | (49.0) | 106 | (65.0) |

LPT, laparotomy; O, omentectomy; Ly, lymphadenectomy; TAH, total abdominal hysterectomy; BSO, bilateral salpingoophorectomy.

by trial. Out of the 914 patients, 62 (6.8%) have been excluded from every analysis, and are not reported in Table II, for one or more of the following reasons: borderline tumour ($n = 4$), unknown FIGO grade ($n = 40$), age ($n = 1$), histotype ($n = 5$) and residual tumour size ($n = 16$). For the remaining 852 patients information was available on the following variables: residual tumour after first surgery, histotype, FIGO stage and grade, age, type of response, center of treatment and type of surgical treatment. Information on performance status (PS) and abdominal cytology was unavailable for respectively 131 and 173 patients. Of the 721 patients for whom information on PS was available, 152 lacked data on abdominal cytology. As described elsewhere (Gruppo Interregionale Cooperativo Oncologico Ginecologia, 1987; Bolis *et al.*, 1980; Sessa *et al.*, 1985; Mangioni *et al.*, 1989), all known prognostic indicators were balanced between treatment groups within each separate trial.

*Evaluation and statistical methods*

Survival is the only end-point considered in this analysis. Death as the only measure of outcome was chosen because this event is unaffected by those time-related biases which may alter end-points such as response or progression-free survival and are almost unavoidable in a retrospective analysis. Time on study or time to death were calculated from the day of first surgery to the cut-off date or to death, if this occurred. Kaplan & Meier (1958) and log-rank test (Tarone & Ware, 1977) methods were used respectively to estimate and compare survival curves (the expected number of events are calculated in the log-rank test under the null-hypothesis).

The Cox model (Cox, 1972) was used for multivariate analysis on survival. Graphic checks of the proportionality assumption were made in all analyses. A significance level of 5% was adopted in all the two-tailed tests.

Prognostic factors taken into account in the step-wise Cox analysis on survival were residual tumour size (<2, 2–5, >5 cm) FIGO stage (III, IV) and grade; cell type (serous, endometrioid + clear cell + mixed, mucinous, undifferentiated, age, centre (Monza, Brescia, others)); type of first surgery (explorative laparotomy, TAH + BSO ± O ± lymphadenectomy, BSO ± O ± lymphadenectomy). Two further sets of analyses were done on the subset of patients for whom information on performance status (PS, Karnofsky index 100–90, <90, $n = 721$) and abdominal cytology (positive, negative, $n = 568$) was also available. The variable treatment was used as a correction factor in Cox regression analysis.

Cut-off date for the analysis was 31 December 1988; the median and the longest follow-up times are respectively 56 and 122 months. There are 31% of patients withdrawn alive (66% in trial 4 and 23% in trials 1–3).

### Results

Table III shows the final results of Cox analysis on the whole population (852 patients) and does not include FIGO grade, centre, and type of surgery because these did not significantly affect the probability of surviving. Age >50 years, stage IV disease, cell type other than serous and tumour size more than 5 cm were associated with a higher probability of death. The risk of dying rose progressively with the size of residual tumours from 1 in the <2 cm category, two-fold for 2–5 cm, and three-fold in the >5 subgroup.

The same analysis was repeated on the subset of 721 patients for whom PS were available (Table III). By adding this variable, tumour size and cell type remained important determinants but the other previously significant factors – stage and age – lost their prognostic value. A good PS (100–90) carried significant benefit. The same anlaysis was repeated in a further reduced subset of 568 cases for whom information was available on abdominal cytology. This variable did not significantly affect survival or change the previous results.

Each subgroup by residual tumour size (<2 cm, 2–5, >5 cm) was further divided according to the remaining prognostic variables identified by Cox analysis – performed on the whole population ($n = 852$) – to be significant (cell type) or borderline significant (age, stage). Six prognostic groups were thus created. In each subgroup, patients featuring the best combination of prognostic characteristics (age <50 years, serous cell type and stage III) were juxtaposed to patients presenting all other combinations.

Table IV sets out the distribution of patients by prognostic class and the relative observed/expected (O/E) ratios, 5-year survival and median survival times (MST). In patients with residual tumour <2 cm, the additional co-factors differentiated between two distinct populations with 5-year survival rates 62% and 41% and median survival times of 108 and 36 months. In the other two sub-groups, 2–5 cm and >5 cm, younger age, lesser stage and presence of serous cell type do not seen to have a relevant impact on the prognostic strength of the tumour size.

Figure 1a and b shows the overall survival curve and the curves for the entire population stratified according to the six prognostic classes.

Figure 2a gives the curves by residual tumour size alone and Figure 2b the curves for the <2 cm population further broken down (microscopic, <1 cm, 1–2 cm). The population with microscopic disease was equally distributed between the two first sub-groups of risk categories (<2 cm, good risk and <2 cm, poor risk).

Table V also sets out the distribution of patients by prognostic class, but utilises the class definition derived from Cox analysis performed on the subset of 721 patients for whom PS was available. The risk classes thus identified are less distinctively differentiated in terms of 5 years survival and median survival time, when compared with those obtained through the first analysis.

Table VI sets out the O/E ratios overall and after stratification by tumour size (<2 cm, 2–5, >5 cm), by trial and by treatment. Three observations can be made when considering the overall O/E ratios: (1) the first trial, comparing

**Table III** Final results of Cox's regression analysis[a]

| *Regression variables*[b] | *All cases (n = 852)* | *Cases with PS (n = 721)* |
|---|---|---|
| *Age (years)* | | |
| <50 | 1 | |
| ⩾50 | 1.01 (1.0–1.1) | n.s. |
| *FIGO stage* | | |
| III | 1 | |
| IV | 1.31 (1.1–1.6) | n.s. |
| *Cell type* | | |
| Serous | 1 | |
| Others | 1.4 (1.2–1.6) | 1.4 (1.2–1.7) |
| *Residual tumour* | | |
| <2 cm | 1 | 1 |
| 2–5 cm | 2.12 (1.6–2.7) | 2.3 (1.7–3.0) |
| >5 cm | 3.15 (2.8–4.4) | 3.2 (2.5–4.0) |
| *Performance status (PS):* | | |
| 90–100 | | 1 |
| >90 | | 1.5 (1.2–1.8) |

[a]Relative risk (95% Cl). [b]Reference level assigned a value of 1.0. The higher the risk, the greater the probability of dying; the lower the risk (i.e. <1), the higher the probability of surviving. n.s. = not significant.

**Table IV** Prognostic groups according to Cox analysis

| | *Prognostic factors* | | | | *Patients* | | | | |
|---|---|---|---|---|---|---|---|---|---|
| *Group* | *RTS*[a] *(cm)* | *Stage* | *Age (years)* | *Cell type* | *No.* | *%* | *O/E*[b] *ratio* | *% 5 years survival* | *MST*[c] *(months)* |
| 1 | <2 | III | <50 | serous | 62 | 7 | 0.32 | 62 | 108 |
| 2 | <2 | any | any | any | 184 | 22 | 0.56 | 41 | 36 |
| 3 | 2–5 | III | <50 | serous | 32 | 4 | 0.97 | 21 | 24 |
| 4 | 2–5 | any | any | any | 140 | 16 | 0.96 | 22 | 20 |
| 5 | >5 | III | <50 | serous | 45 | 5 | 1.18 | 14 | 18 |
| 6 | >5 | any | any | any | 389 | 46 | 1.64 | 7 | 15 |

[a]Residual tumour size; [b]observed/expected; [c]median survival time.

**Figure 1** a, Overall survival. b, Survival by risk-group (see Table IV for risk group definition). ● group 1, ▲ group 2, ■ group 3, ○ group 4, □ group 5, △ group 6.

**Figure 2** a, Survival by residual tumour size after first surgery: ● <2 cm, ▲ 2–5 cm, ■ >5 cm. b, Survival by residual tumour size after first surgery: ● absent or microscopic, ▲ <1 cm, ■ ⩾1 cm.

single agent cyclophosphamide and the combinations with adriamycin (AC) shows the highest O/E ratio; (2) the addition of a third active drug to the AC combination (i.e. cisplatin or hexamethylmelamine) reduced the O/E to the unity; and (3) the latest trial comparing single agent high-dose (100 mg) cisplatin to carboplatin shows the lowest O/E ratio. The same pattern was evident after stratification by residual tumour size.

When the O/E ratios within each separate trial are considered, the pattern is the same as for the overall O/E, with no major differences between treatment arms, although in trial no. 3 low-dose (50 mg $m^{-2}$) cisplatin alone arm (CAP *vs* CP *vs* P) had an O/E of 1.20 compared to ratios below unity for the two combination arms (CAP and CP).

Figure 3 is the graphic correspondent of Table VI, showing the survival curves for each trial. The AC/C trial is doing significantly worse, the high-dose cisplatin/carboplatin arm shows better survival, although with a much shorter follow-up, while the curves for HAC/PAC and CAP/CP/P trials are superimposed and in an intermediate position. Survival curves stratified by residual tumour size are shown in Figure 4a (<2 cm), b (2–5 cm) and c (>5 cm). In the subgroup of 246 patients with residual tumour size below 2 cm the survival curves for the four trials are completely superimposed and the worse survival experience observed for the whole population in trial no. 1 (C *vs* AC) is no longer detectable

**Table V** Prognostic factors according to Cox analysis including performance status (PS)

| | *Prognostic factors* | | | *Patients* | | | | |
|---|---|---|---|---|---|---|---|---|
| *Group* | *RTS*[a] *(cm)* | *PS* | *Cell type* | *No.* | *%* | *O/E*[b] *ratio* | *% 5 years survival* | *MST*[c] *(months)* |
| 1 | <2 | >90 | serous | 101 | 14 | 0.40 | 50 | 43 |
| 2 | <2 | any | any | 104 | 14 | 0.58 | 36 | 32 |
| 3 | 2–5 | >90 | serous | 71 | 10 | 0.75 | 28 | 21 |
| 4 | 2–5 | any | any | 70 | 10 | 1.41 | 17 | 7 |
| 5 | >5 | >90 | serous | 151 | 21 | 1.22 | 19 | 7 |
| 6 | >5 | any | any | 224 | 31 | 1.75 | 14 | 5 |

[a]Residual tumour size; [b]observed/expected; [c]median survival time.

**Figure 3** Survival by trial: ● trial 1-AC/C, ▲ trial 2 HAC/PAC, ■ trial 3 PAC/CP/P, O trial 4 carboplatin/P 100.

**Table VI** Observed/expected ratio by trial and treatment

| *Trial no.* | *O/E ratio* | *Arms* | *O/E ratio* | *No. of pts* | *No. of events/trial* |
|---|---|---|---|---|---|
| | | C | 1.47 (1.88) | | |
| 1 | 1.54 (1.91) | | | 63 | 47 |
| | | AC | 1.67 (1.95) | | |
| | | HAC | 1.05 (1.03) | | |
| 2 | 0.95 (0.94) | | | 107 | 84 |
| | | PAC | 0.89 (0.94) | | |
| | | CAP | 0.89 (0.86) | | |
| 3 | 1.02 (1.03) | CP | 0.98 (0.94) | 529 | 399 |
| | | P | 1.20 (1.23) | | |
| | | P100 | 0.61 (0.54) | | |
| 4 | 0.73 (0.66) | | | 163 | 55 |
| | | JM8 | 0.88 (0.81) | | |

In parenthesis is the O/E after stratification by residual tumour size (⩽2 cm, 2–5, >5 cm).

(Figure 4a). However, the pattern of a marked difference between trial 1 and all the others, and with the caveat of the shorter follow-up, the superiority of trial 4, reappears when plotting the survival curves for the >2 cm sub-groups (Figure 4b, 2–5 cm; Figure 4c, >5 cm).

### Discussion

The aim of this paper was a critical analysis of the results from four randomised clinical trials, inclusive of more than 850 patients, conducted by a single cooperative group in advanced epithelial ovarian cancer in the last decade. There were several purposes for the analysis: (1) to determine the impact of selected prognostic variables on survival of advanced ovarian cancer patients; (2) to obtain, from the interaction of favourable prognostic factors and treatment, an approximative estimate of the magnitude of the survival advantage associated with the use of platinum-based combination chemotherapy; and (3) to establish the implications of these two points for clinical research and ovarian cancer patients care.

### *Impact of selected prognostic variables on survival of advanced ovarian cancer patients*

Residual tumour size is the major determinant of survival. As defined by the multivariate analysis, the addition of age, stage and histology further identify a sub-population within the less than 2 cm subgroup, with a better prognosis (Table IV). However, this subgroup comprises less than 10% of the overall population and its survival curve does not seem to have reached a plateau even after 5 years. For patients with residual tumour in excess of 2 cm addition of the other prognostic variables fails to discriminate among subgroups

**Figure 4** **a**, Survival by trial in patient with residual tumour size after first surgery <2 cm: ● trial 1 AC/C, ▲ trial 2 HAC/PAC, ■ trial 3 PAC/CP/P, O trial 4 carboplatin/P. **b**, Survival by trial in patients with residual tumour size after first surgery 2–5 cm: ● trial 1 AC/C, ▲ trial 2 HAC/PAC, ■ trial 3 PAC/CP/P, O trial 4 carboplatin/P. **c**, Survival by trial in patients with residual tumour size after first surgery >5 cm: ● trial 1 AC/C, ▲ trial 2 HAC/PAC, ■ trial 3 PAC/CP/P, O trial 4 carboplatin/P.

with markedly different survival experience (Figure 1b).

PS was another strong independent factor affecting survival. The addition of this variable in the Cox model yielded a 1.5 relative risk of dying for patients with a PS score below 90, while nullifying the influence of stage and age. This is not surprising since PS could be a 'comprehensive' marker of the same relationship between the patient's status and the extent of disease expressed by the combination of stage and age in the first model, despite the different nuances.

While all published papers on multivariate analysis agree on the importance of residual tumour as a prognostic determinant, the prognostic importance of all other variables varied from one study to another (Griffiths, 1975; Swenerton *et al.*, 1985; Redman *et al.*, 1986; Gruppo Interregionale Cooperativo Oncologico Ginecologia, 1987; Neijt *et al.*, 1984, 1987). This may be accounted for by both the small populations studied (in all cases fewer than 200 patients were analysed) and the different 'cocktails' of factors considered (several included weight loss, other excluded age and/or stage, etc). The strength of this study is the large sample on which results are based. However, the relevance of prognostic factors other then residual tumour size should be further tested to avoid biases inherent to retrospective and subgroup analysis. The results presented in Table IV and Figure 1b need to be prospectively validated on an independent data set.

*Approximate magnitude of the survival advantage associated with platinum-based combination chemotherapy in ovarian cancer*

The overall 5-year survival rate in advanced epithelial cancer in this series of 852 patients treated between 1978 and 1987 is twice that reported historically (Richardson *et al.*, 1985) for 5,254 cases collected from 1973 to 1974 (22.1, 95% CL 18.7–25.4 *vs* 10.4%). In addition the 1, 2 and 3 year survival figures are practically the same as in the most recent trials utilising aggressive polychemotherapies like the CHAP-5 regimen (Neijt *et al.*, 1984, 1987).

Since we pooled data from four consecutive trials, in the multivariate analysis we considered type of treatment only as a correcting factor for determining the influence of the various prognostic variables on survival. However, survival was also influenced by treatment or at least by type of strategy, as can be seen from Figure 3 and Table VI. This observation must be tempered in consideration of the historical nature of our analysis which can be biased by changes in the characteristics of the treated groups and in the types of ancillary treatment and/or diagnostic work-up (Table II suggests a worsening of the patients over time, in spite of a higher incidence of more aggressive surgery). That not withstanding, patients treated with the earlier strategic approach, single agent cyclophosphamide or a combination of cyclophosphamide and adriamycin did worse than all the others. Patients exposed to cisplatin – either alone at high-doses (100 mg $m^2$) or in combination (CP, CAP) at low-doses (50 mg $m^2$) – did better, but the HAC treated patients did no worse than the previous category. However, if platinum-based chemotherapies – either single agent or combinations – seem to have prolonged the median survival time in advanced ovarian cancer patients, the fraction of long-term survivors has not increased markedly, especially in the sub-group most susceptible of being cured (i.e. patients with less than 2 cm residual tumour, Figure 3a). In fact, results from randomised trials, our own (Gruppo Interregionale Cooperativo Oncologico Ginecologia, 1987) and more recent ones (Neijt *et al.*, 1987; Omura *et al.*, 1983; Tomirotti *et al.*, 1988), suggest that if a difference exist between less aggressive regimens (cisplatin, cisplatin + cytoxan) and CAP is of an order of magnitude much lower than that hoped for in the early 1980s (i.e. less than 20%).

*Implications for clinical research and care in ovarian cancer*

Prognostic factors are sought not only to help understand the natural history of a disease but also for more 'decisional' purposes in both clinical practice and research.

In clinical practice, the knowledge that advanced ovarian cancer patients can be assigned to groups with distinct prognostic characteristics may serve doctors as a guideline for a more accurate estimate of the trade-offs between toxicity and survival offered by chemotherapy.

In clinical research, prognostic factors are often used for adjusting for randomisation imbalances in the analysis/interpretation of clinical trial results. This work suggests there is great heterogeneity – in terms of survival probabilities – within an apparently homogeneous population, historically labelled as 'advanced disease'. The difference in survival between the best-prognosis group and all the others is impressive. It follows that the results of any trial could quite dramatically change – and independently from the real impact of whatever experimental treatment was tested – depending on the size of this fraction of patients. Since generally not more than 20% of the patient population for a given tumour type, including ovarian, seen at any given institution (Wittes & Friesman, 1988), enters controlled clinical trials, this kind of selection bias could conceivably play a role in up-grading (or down-grading) the final results of the trial itself.

This might partially explain the different results obtained by different centres/investigators utilising similar regimens in advanced ovarian cancer (Tomirotti *et al.*, 1988; Neijt *et al.*, 1987; Decker *et al.*, 1982; Bell *et al.*, 1982; Conte *et al.*, 1986; Williams *et al.*, 1985; Young *et al.*, 1978; Bertelsen *et al.*, 1987; Wilbur *et al.*, 1987; Carmo-Pereira *et al.*, 1983; Omura *et al.*, 1986). It also strikes a point against historical comparisons in which the lack of randomisation is even more likely to unbalance the prognostic subgroups and consequently introduce powerful biases in the conclusions.

To facilitate international communication of data, common criteria for defining and reporting risk groups in this disease should perhaps be developed and agreed upon, as has been done for other diseases (Mastrangelo *et al.*, 1986).

The second implication for clinical research stems from the recognition that the differences in survival among the various prognostic groups, although impressive, are quantitative, not qualitative. In other world the 'kinetics' of survival of the best and worst risk groups are similar. Thus the current prognostic factors are useless for selecting a 'biologically' different sub-population, as was done, for example, within the acute leukaemias for the B immunophenotype. The current prognostic factors in ovarian cancer probably represent a too remote epiphenomenon(a) of the underlying abnormality(ies) to be of use for this purpose.

Finally, no striking differences were observed in the long-term results with eight different mono or polychemotherapy regimens. The results of other randomised clinical trials in the past decade point in the same direction (Gruppo Interregionale Cooperativo Oncologico Ginecologia, 1987; GGCOSA, 1986; Bell *et al.*, 1982; Williams *et al.*, 1985; Dembo, 1986; Burslem & Wilkinson, 1986). They suggest a superiority in terms of response and progression-free disease of combination chemotherapy over single-agent alkylators or cisplatin, but are ambiguous in terms of survival and cost/benefit ratios possibly because much larger sample sizes are needed to detect small survival differences (i.e. 10% or less).

The implications are two-fold: first that clinical research in this disease has reached a plateau phase, and secondly that efforts should be directed beyond the repetitive area of comparing 'new' combinations of old drugs. Perhaps while awaiting new drugs or truly innovative new ideas, clinical research in this area should tackle the fact that although cisplatin-based chemotherapy seems to play an important role, we are far from having established any universally acceptable standard.

We are deeply indebted to the following clinicians who participated in the trials: P.F. Bolis, G. Bortolozzi, G. Giardina, G. Mangili, P. Meroni, N. Natale, A. Paganelli, L. Redaelli, U. Redaelli, P. Sanpaolo, M. Santoiemma and F. Vergadoro and to A. Liberati for fruitful discussion. The generous contribution of the Italian Association for Cancer Research, Milan, Italy, is gratefully acknowledged. Partially supported by Consiglio Nazionale delle Ricerche (CNR) and by Regione Lombardia.

### References

BELL, D.R., WOODS, R.L., LEVI, J.A. & 2 others (1982). Advanced ovarian cancer: a prospective randomised trial of chlorambucil versus combined cyclophosphamide and cis-diamminedichloroplatinum. *Aust. NZ J. Med.*, **12**, 245.

BERTELSEN, K., JAKOBSEN, A., ANDERSEN, J.E. & 21 others (1987). A randomized study of cyclophosphamide and cis-platinum with or without doxorubicin in advanced ovarian carcinoma. *Gynecol. Oncol.*, **28**, 161.

BOLIS, G., BORTOLOZZI, G., CARINELLI, G. & 4 others (1980). Low-dose cyclophosphamide versus adriamycin plus cyclophosphamide in advanced ovarian cancer. *Cancer Chemother. Pharmacol.*, **4**, 129.

BURSLEM, R.W. & WILKINSON, P.M. (1986). Treating ovarian cancer. *Br. Med. J.*, **293**, 972.

CARMO-PEREIRA, J., COSTA, F.O. & HENRIQUES, E. (1983). Cis-platinum, adriamycin, and hexamethylmelamine versus cyclophosphamide in advanced ovarian carcinoma. *Cancer Chemother. Pharmacol.*, **10**, 100.

CISLAGHI, C., DECARLI, A., LA VECCHIA, C., LA VERDA, N., MEZZANOTTE, G. & SMANS, M. (1986). *Data Statistics and Maps on Cancer Mortality: Italia 1975/1977*. Pitagora Editrice: Bologna.

CONTE, P.F., BRUZZONE, M., CHIARA, S. & 31 others (1986). A randomized trial comparing cisplatin plus cyclophosphamide versus cisplatin, doxorubicin, and cyclophosphamide in advanced ovarian cancer. *J. Clin. Oncol.*, **4**, 965.

COX, D.R. (1972). Regression models and life tables. *J. R. Stat. Soc. B*, **34**, 187.

DECKER, D.G., FLEMING, T.R., MALKASIAN, G.D. Jr & 3 others (1982). Cyclophosphamide plus cis-platinum in combination: treatment program for stage III or IV Ovarian carcinoma. *Obstet. Gynecol.*, **60**, 481.

DEMBO, A.J. (1986). Controversy over combination chemotherapy in advanced ovarian cancer: what we learn from reports of matured data. *J. Clin. Oncol.*, **4**, 1573.

GRIFFITHS, C.T. (1975). Surgical resection of tumor bulk in the primary treatment of ovarian carcinoma. *Natl Cancer Inst. Monogr.*, **42**, 101.

GRUPPO INTERREGIONALE COOPERATIVO ONCOLOGICO GINECOLOGIA (1987). Randomised comparison of cisplatin with cyclophosphamide/cisplatin and with cyclophosphamide/doxorubicin/cisplatin in advanced ovarian cancer. *Lancet*, **ii**, 353.

GYNAECOLOGICAL GROUP, CLINICAL ONCOLOGICAL SOCIETY OF AUSTRALIA, AND THE SIDNEY BRANCH, LUDWIG INSTITUTE FOR CANCER RESEARCH (1986). Chemotherapy of advanced ovarian adenocarcinoma: a randomized comparison of combination versus sequential therapy using chlorambucil and cisplatin. *Gynecol. Oncol.*, **23**, 1.

KAPLAN, E.L. & MEIER, P. (1958). Non parametric estimation from incomplete observations. *J. Am. Stat. Assoc.*, **53**, 457.

MANGIONI, C., BOLIS, G., PECORELLI, S. & 10 others (1989). Randomized trial comparing cisplatin and carboplatin. *J. Natl Cancer Inst.*, **81**, 1464.

MASTRANGELO, R., POPLACK, D., BLEYER, A., RICCARDI, R., SATHER, H. & D'ANGIO, G. (1986). Report and recommendations of the Rome Workshop concerning poor-prognosis acute lymphoblastic leukemia in children: biologic bases for staging, stratification, and treatment. *Med. Pediatr. Oncol.*, **14**, 191.

NEIJT, J.P., TEN BOKKEL HUININK, W.W., VAN DER BURG, M. & 8 others (1984). Randomised trial comparing two combination chemotherapy regimens (Hexa-CAF vs CHAP-5) in advanced ovarian carcinoma. *Lancet*, **ii**, 594.

NEIJT, J.P., TEN BOKKEL HUININK, W.W., VAN DER BURG, M. & 8 others (1987). Randomized trial comparing two combination chemotherapy regimens (CHAP-5 v CP) in advanced ovarian carcinoma. *J. Clin. Oncol.*, **5**, 1157.

OMURA, G., BLESSING, J.A., EHRLICH, C.E. & 4 others (1986). A randomized trial of cyclophosphamide and doxorubicin with or without cisplatin in advanced ovarian carcinoma. A Gynecologic Oncology Group study. *Cancer*, **57**, 1725.

OMURA, G.A., MURROW, C.P., BLESSING, J.A. & 4 others (1983). A randomized comparison of melphalan versus malphalan plus hexamethylmelamine in ovarian carcinoma. *Cancer*, **51**, 783.

REDMAN, J.R., PETRONI, G.R., SAIGO, P.E., GELLER, N.L. & HAKES, T.B. (1986). Prognostic factors in advanced ovarian carcinoma. *J. Clin. Oncol.*, **4**, 515.

RICHARDSON, G.S., SCULLY, R.E., NIR KUI, N. & NELSON, J.H. Jr. (1985). Common epithelial cancer of the ovary (part I). *N. Engl. J. Med.*, **312**, 415.

SESSA, C., BOLIS, G., COLOMBO, N. & 4 others (1985). Hexamethylmelamine, adriamycin, and cyclophosphamide (HAC) versus cisdichlorodiamineplatinum, adriamycin, and cyclophosphamide (PAC) in advanced ovarian cancer: a radomized clinical trial. *Cancer Chemother. Pharmacol.*, **14**, 222.

SWENERTON, K.D., HISLOP, T.G., SPINELLI, J., LERICHE, J.C., YANG, N. & BOYES, D.A. (1985). Ovarian carcinoma: a multivariate analysis of prognostic factors. *Obstet. Gynecol.*, **65**, 264.

TARONE, R.E. & WARE, J. (1977). On distribution-free tests for equality of survival distributions. *Biometrika*, **64**, 156.

TOMIROTTI, M., PERRONE, S. GIÈ, P. & 8 others (1988). Cisplatin (P) versus cyclophosphamide, adriamycin and cisplatin (CAP) for stage III-IV epithelial ovarian carcinoma: a prospective randomized trial. *Tumori*, **74**, 573.

WILBUR, D.W., RENTSCHLER, R.E., WAGNER, R.J., KEENEY, E.D., KING, A. & HILLIARD, D.A. (1987). Randomized trial of the addition of cis-platin (DDP) and/or BCG to cyclophosphamide (CTX) chemotherapy for ovarian carcinoma. *J. Surg. Oncol.*, **34**, 165.

WILLIAMS, C.J., MEAD, G.M., MACBETH, F.R. & 8 others (1985). Cisplatin combination chemotherapy versus chlorambucil in advanced ovarian carcinoma: mature results of a randomized trial. *J. Clin. Oncol.*, **3**, 1455.

WITTES, R.E. & FRIEDMAN, M.A. (1988). Accrual to clinical trials. *J. Natl Cancer Inst.*, **80**, 884.

YOUNG, R.C., CHABNER, B.A., HUBBARD, S.P. & 6 others (1978). Advanced ovarian adenocarcinoma. A prospective clinical trial of melphalan (L-PAM) versus combination chemotherapy. *N. Engl. J. Med.*, **299**, 1261.